# EDTA-induced remobilization of lead from suspended particulate matter in contaminated water samples from the Innerste River: a statistical evaluation

**DOI:** 10.1007/s11356-026-37480-x

**Published:** 2026-02-07

**Authors:** Jan Klaus Hinrichs, Markus Herrmann, Aaron Bauer, Dieter Steffen

**Affiliations:** Department of Chemistry, Institute of Biology and Chemistry, University of Hildesheim, Universitätsplatz 1, 31141 Hildesheim, Germany

**Keywords:** EDTA, Lead remobilization, Suspended particulate matter, Speciation modeling, Bayesian statistics, Environmental risk assessment

## Abstract

Ethylenediaminetetraacetic acid (EDTA), extensively used across multiple industries, has long been discussed for its potential to enhance heavy metal mobility in aquatic systems, with studies yielding contradictory results. This study examines the remobilization of particle-bound lead from suspended particulate matter (SPM) in the Innerste River (Lower Saxony, Germany), which is affected by historical mining and known for substantial Pb contamination. Using real river water containing its native SPM to preserve the chemical matrix of the system, we assessed Pb partitioning between total and dissolved phases to evaluate EDTA’s remobilization potential. Baseline dissolved lead concentrations reached up to 1.8 µg L^−1^ (median 0.69 µg L^−1^). Across all batch experiments, a measurable increase in the dissolved Pb fraction occurred only at EDTA concentrations far exceeding those measured in the river (0.68–3.8 µg L^−1^). Bayesian concentration–response modelling yielded no-effect concentrations (NEC) between 210 and 530 µg L^−1^. Complementary speciation modelling showed that shifts in Pb speciation occur only at EDTA concentrations near the experimentally derived NEC values. These findings show that current EDTA concentrations in the Innerste are unlikely to remobilize Pb from SPM. The study also provides a statistically supported NEC estimate based on batch experiments using unaltered river water containing its naturally present SPM. To our knowledge, this is the first application of Bayesian NEC modelling to EDTA-induced Pb remobilization.

**Supplementary Information:**

The online version contains supplementary material available at 10.1007/s11356-026-37480-x.

## Introduction

Ethylenediaminetetraacetic acid (EDTA) and its salts are widely used anthropogenic chelating agents applied across multiple industrial sectors, including pulp and paper production (Goto et al. 2023), food preservation (Allen 2015), and upstream oil and gas operations (Hassan et al. 2020), where they bind undesirable metal ions. EDTA-based materials are also used in water treatment, for example, as adsorbents for removing metal ions from wastewater (Zhang et al. 2021). Moreover, EDTA and related complexing agents have been extensively studied in soil remediation (Chen et al. 2022) and phytoremediation (Kamal et al. 2023; Shahid et al. 2014), where they function as soil washing agents and can enhance plant uptake of heavy metals.

Because EDTA is biologically recalcitrant under conventional conditions (Nörtemann 1999) and only partially removed in wastewater treatment plants (WWTP) (Alder et al. 1990; Kari and Giger 1996), it is frequently detected in municipal effluents and surface waters. In a European survey of 13 wastewater samples from eight WWTPs, EDTA showed the highest median concentration (60 µg L^−1^) among 36 polar analytes (Reemtsma et al. 2006). Sector-specific inputs can further elevate levels. Effluents influenced by dairy-processing facilities contained 164–547 µg L^−1^ EDTA, compared with 41–124 µg L^−1^ in effluents receiving domestic wastewater only (Constantino et al. 2017). The Swiss Micropoll study similarly reported EDTA as a ubiquitous microcontaminant, with an average of 20.9 µg L^−1^ in WWTP effluents and 2.8 µg L^−1^ in 202 of 248 surface-water samples (Abegglen and Siegrist 2012).

Studies of the Swiss river Glatt showed that EDTA in surface waters predominantly occurs as Fe(III)– and Zn(II)–EDTA complexes (Bucheli-Witschel and Egli 2001). Similarly, investigations of the inflow and outflow of Swiss wastewater treatment plants demonstrated that EDTA is mainly present as complexes with Fe^3+^, Ni^2+^, or Ca^2+^ (Kari and Giger 1996; Nirel et al. 1998). In natural waters, degradation proceeds primarily via abiotic pathways, particularly through photolytically induced decarboxylation of Fe(III)–EDTA complexes (Nowack 2002).

Ecotoxicological data show that EDTA exhibits low acute and chronic toxicity, with effect thresholds typically in the mg L^−1^ range. In natural waters, however, EDTA occurs almost entirely as metal complexes, and its environmental relevance is largely speciation-driven. By strongly binding both essential trace elements (e.g., Fe, Zn) and toxic metals (e.g., Cu, Cd), EDTA can alter metal bioavailability and, depending on geochemical conditions, either reduce free-ion activities or promote the remobilization of small amounts of bioavailable metals (Schmidt and Brauch 2004).

**Fig. 1:**
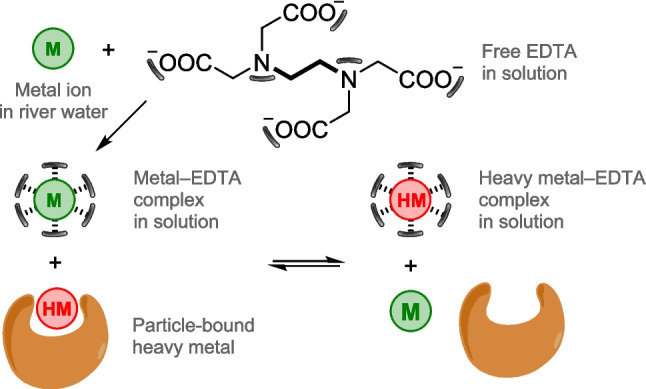
Simplified scheme illustrating EDTA-mediated metal–particle interactions and heavy metal remobilization in river water

Beyond the ecotoxicological aspects, a central point of debate concerns whether environmentally relevant concentrations of EDTA are sufficient to remobilize heavy metals (HM) from contaminated solid phases (e.g., soil, sediment, or suspended solids). In river water, EDTA occurs predominantly as metal–EDTA complexes (M = Fe, Zn, Ca, Mn) (Nowack et al. 1997), and any displacement of particle-bound metals depends on the relative stability constants of the competing metal–EDTA species (Fig. 1).

The partitioning of heavy metals between solid phases and the aqueous phase is governed by factors such as solid composition, metal concentration and speciation, solid-to-solution ratio, contact time, and pH. These controls are well established for soils (Peng et al. 2018; Qi et al. 2025; Rieuwerts et al. 1998) and are similarly relevant for other particulate phases such as sediments and suspended solids. Natural ligands (e.g., humic substances) and anthropogenic chelators such as EDTA further influence metal distribution by competing for dissolved and particle-bound metals. In soils, EDTA enhances metal mobility by forming stable metal–EDTA complexes and thereby limiting adsorption to solid phases; complexation can also promote desorption of particle-bound metals. The stability of metal–EDTA complexes depends on the specific metal and pH, with maximum stability typically observed under slightly acidic to neutral conditions (Bradl 2004).

EDTA’s strong metal-solubilizing capacity is widely exploited in soil remediation (Chen et al. 2022; Lebrun et al. 2023), typically at millimolar concentrations far above those in natural waters. At such low environmental levels, and under heterogeneous geochemical conditions, reported effects of EDTA on metal remobilization range from negligible to pronounced. This span is illustrated by two contrasting examples: Gonsior et al. (1997) reported no detectable remobilization at environmentally relevant EDTA concentrations, whereas Jiann et al. (2013) found that elevated EDTA levels in polluted rivers were associated with increased dissolved trace-metal fractions.

In rivers where solid phases (e.g., sediments or suspended particles) are contaminated with heavy metals, their potential remobilization into the dissolved phase is of particular concern. One such system is the Innerste River (Lower Saxony, Germany), which remains heavily affected by legacy mining in the Harz Mountains, with lead as a dominant pollutant (Ludolphy et al. 2022; Steingräber et al. 2022). Most Pb is transported in particulate form, with annual loads of 16.82 t year^−1^ in the particulate phase vs. 0.65 t year^−1^ in the dissolved phase (Schaffer 2019). The dissolved fraction is critical for compliance with the European Water Framework Directive, which specifies an annual average environmental quality standard (AA-EQS) of 1.2 µg L^−1^ for inland surface waters (Official Journal of the European Union, 2013) . Anthropogenic chelators such as EDTA may increase dissolved Pb. However, whether environmentally relevant concentrations can remobilize particle-bound metals remains debated and has not yet been assessed for the Innerste.

To address this, we quantified the EDTA concentration at which remobilization of heavy metals into the dissolved phase becomes detectable. We focused on suspended particulate matter (SPM), capturing particle–water interactions under conditions representative of the water column, in contrast to sediment-based studies that rely on much higher solid-to-liquid ratios (Bordas and Bourg 1998; Soltan 2006). Experiments were conducted using real river water to preserve the natural SPM load and solid-to-liquid ratio of the Innerste. The resulting concentration–response relationship was analysed using the no-effect concentration (NEC), a model-based threshold metric formalized by Pires et al. (2002) and later advanced in Bayesian frameworks by Fox et al. (Fisher and Fox 2023; Fox 2010). To our knowledge, this is the first NEC-derived estimate of EDTA-induced heavy metal remobilization in a real river system under in-situ SPM conditions.

## Materials and methods

### Sample collection sites

**Fig. 2:**
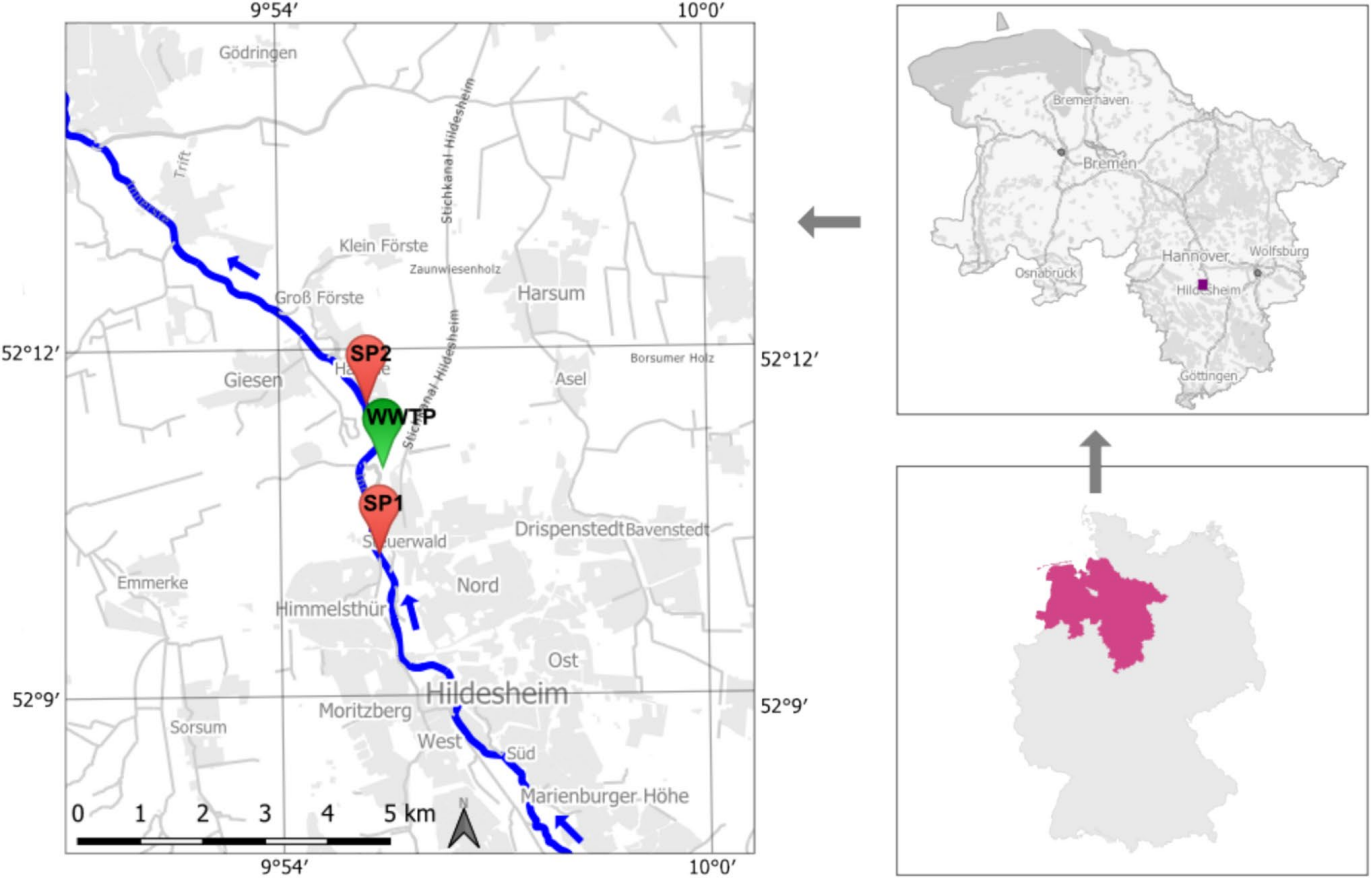
Locations of sampling sites SP1 (upstream of the WWTP discharge) and SP2 (downstream) along the Innerste River in Hildesheim, Germany

Two sampling sites along the Innerste River (Hildesheim district, Lower Saxony, Germany) were selected to assess the influence of effluent discharge on baseline concentrations of lead and EDTA and on site-specific NEC values derived from the remobilization experiments (Fig. 2). The upstream site (SP1) is located at a pedestrian bridge near Steuerwald Castle (52.168199° N, 9.925149° E; WGS84) and represents river conditions unaffected by the Hildesheim WWTP. The downstream site (SP2) is situated at the Lendertberg Bridge in the village of Hasede (52.192150° N, 9.920061° E; WGS84), downstream of the WWTP discharge, and was selected to capture potential effluent-related changes in baseline concentrations and remobilization behavior.

### Remobilization experiments

At each sampling site, approximately 10 L of river water was collected mid-stream and just below the surface using a clean container (grab sampling) and transferred to a pre-cleaned 10 L HDPE carboy for transport to the laboratory. To maintain homogeneity and prevent settling of suspended particles, the bulk samples were thoroughly shaken before further processing. Immediately afterward, 100 mL aliquots were dispensed into 250 mL polyethylene (PE) screw-cap containers using a volumetric pipette.

For EDTA spiking, two stock solutions were used depending on the target concentration. For additions up to 100 µg L^−1^, a 10 mg L^−1^ EDTA stock solution was used to minimize the added volume. For higher additions (≥ 200 µg L^−1^), a 1000 mg L^−1^ stock solution was applied, enabling spiking up to 4000 µg L^−1^ with minimal dilution of the river water samples. Each EDTA spiking level was represented by one aliquot (*n* = 1).

After spiking, all subsamples were shaken simultaneously on an orbital shaker (Bühler KL 2) to ensure identical treatment conditions and to avoid sequential handling or potential aging effects. The batch experiments were conducted at room temperature (20–22 °C) with gentle, continuous mixing at 100 rpm. This shaking intensity was sufficient to keep suspended particles homogenously distributed without causing excessive turbulence or splashing, thereby approximating moderate hydrodynamic agitation under reproducible laboratory conditions. A shaking time of 2 h was selected based on literature indicating that lead remobilization typically occurs within the first 3 h in the presence of EDTA (Parsadoust et al. 2022). In some experiments, the shaking time was extended to 24 h to assess potential effects of prolonged particle–EDTA contact in river water.

For each subsample in the remobilization series, 10 mL was withdrawn from the 100 mL aliquots and filtered through a 0.45 µm cellulose acetate syringe filter into 50 mL centrifuge tubes for dissolved lead analysis. Additionally, 2 mL of the unfiltered whole-water subsample (containing both dissolved and particulate fractions) was transferred directly into autosampler vials for total lead determination. Both filtered and unfiltered subsamples were stabilized by adding concentrated nitric acid (HNO_3_, 65%) corresponding to 0.5% of the sample volume (50 µL to 10 mL filtered aliquots; 450 µL to 90 mL unfiltered aliquots).

**Fig. 3:**
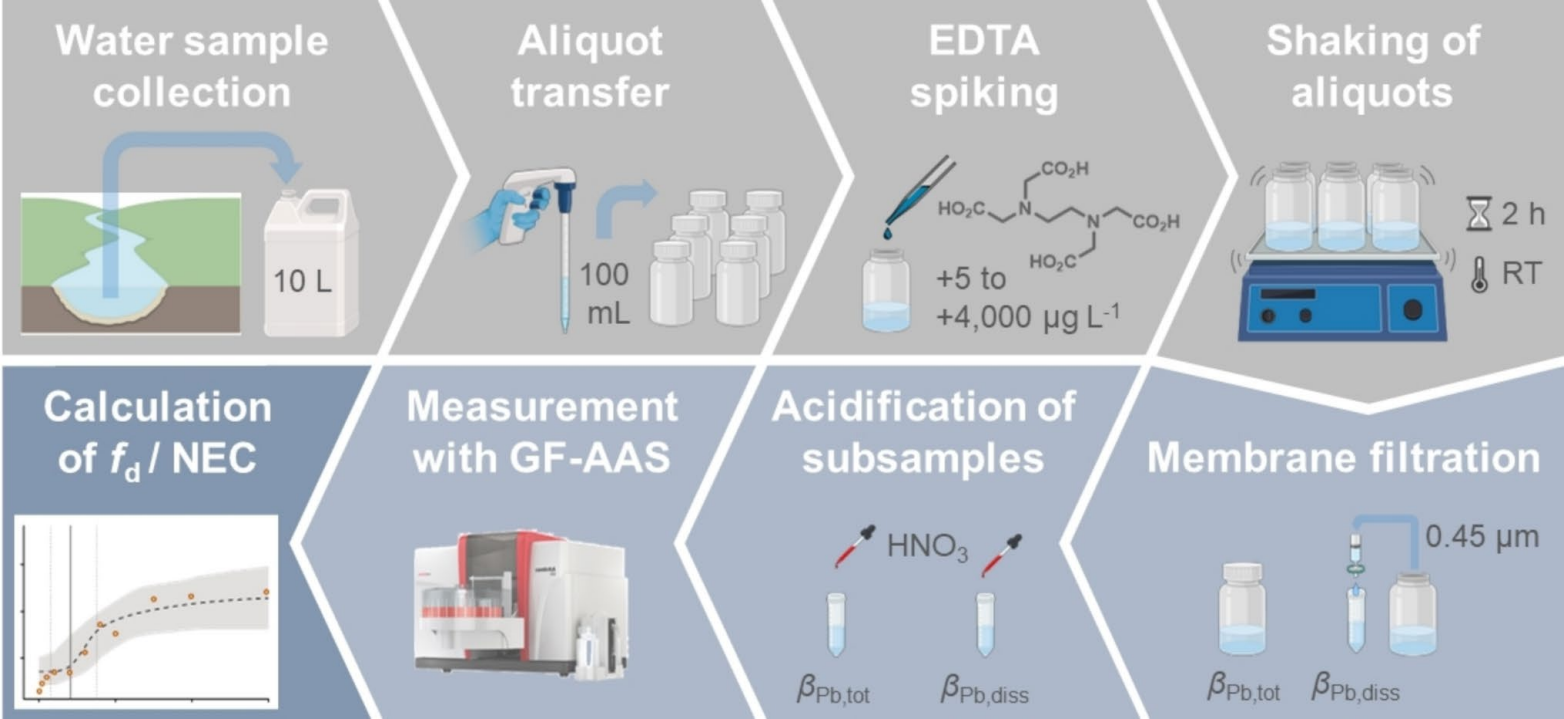
Schematic workflow of the EDTA remobilization experiments in river water. This figure was created with BioRender

For a subset of samples, an exploratory aqua regia microwave digestion was conducted in accordance with DIN EN ISO 15587-1 (2002). The digestion was performed using a MARSXpress microwave system (CEM). Briefly, 25.0 mL of sample was transferred into Teflon digestion vessels and mixed with 6.0 mL concentrated HCl and 2.0 mL concentrated HNO_3_. The vessels were sealed and heated to 175 °C for 10 min. After cooling, the digests were filtered (Whatman 595 1/2) into 50 mL volumetric flasks and diluted to volume with Milli-Q water. All reagents were of analytical grade. Lead concentrations were determined using graphite furnace atomic absorption spectroscopy (GF-AAS). A schematic overview of the experimental procedure is shown in Fig. 3.

SPM content was determined gravimetrically according to DIN 38409-2 (1987) for a subset of samples. Depending on the particulate load, between 500 and 2000 mL of sample were vacuum-filtered through pre-rinsed and dried glass fiber filters (Macherey–Nagel MN 85/70 BF). The filters were then dried at 105 °C, cooled to room temperature in a desiccator, and immediately weighed. SPM concentrations were calculated from the mass increase of the filters relative to the filtered sample volume.

### Analytical methods and instrumentation

#### Gas chromatographic determination of EDTA

The initial EDTA content of the river water samples was determined using the standardized gas chromatographic method DIN EN ISO 16588 (2004), with minor adaptations to suit experimental conditions. Initially, 100 mL of each water sample was spiked with 200 µL of the internal standard 1,2-propylenediaminetetraacetic acid (1,2-PDTA, 10 mg L^−1^) in a wide-neck Erlenmeyer flask. The aqueous solution was evaporated to dryness in an oven at 105 °C, and the residue was transferred with 10 mL of hydrochloric acid (1 M) into PTFE reaction vessels. The solution was again evaporated to dryness on a heating block. The dried residue was then reacted with 2 mL of an esterification reagent (1-propanol:acetyl chloride, 9:1 v/v) for 3.5 h at 90 °C in a sealed Teflon vessel. After cooling, the reaction mixture was transferred to a 50 mL volumetric flask and diluted to the mark with a potassium hydrogen carbonate solution (25% w/w). The aqueous solution was extracted with 1.5 mL of n-hexane, and the organic phase was dried over sodium sulfate.

Gas chromatographic analysis of the organic extracts was performed using a GC 2010 Plus gas chromatograph (Shimadzu) with an automatic injector (AOC 20i), a split/splitless injector, and a nitrogen-phosphorus selective flame thermionic detector (FTD 2010 Plus). The system utilized an Rtx 5 capillary column (Restek) as the stationary phase, helium (purity 5.0, Westfalen) as the carrier gas, and purified compressed air (Parker Balston Model 75–83) along with hydrogen (5.0, Linde) as detector fuel gases.

Calibration was performed using EDTA standard solutions with 1,2-PDTA as the internal standard. Quality control included calibration checks at defined EDTA concentrations, procedural blanks, and continuous verification of detector response. The internal standard corrected for potential losses during sample preparation and ensured consistent detector performance. Limits of detection (LOD) and quantification (LOQ) were calculated according to DIN 32645 (2008), yielding values of 0.19 µg L^−1^ and 0.70 µg L^−1^, respectively. The standardized GC method is well established for determining complexing agents in natural waters and provides highly stable calibration and instrument performance. For this reason, single determinations per EDTA concentration and sampling site were considered sufficient to generate reliable data.

#### Determination of lead using atomic absorption spectroscopy (AAS)

Lead concentrations in the filtered and unfiltered subsamples were determined using a high-resolution continuum-source atomic absorption spectrometer (HR-CS-AAS, contrAA 800D; Analytik Jena) equipped with a graphite furnace and an automatic sampler (MPE 60). A xenon short-arc lamp and a CCD detector provided high spectral resolution and accuracy for lead quantification across the studied concentration range. Calibration was performed with aqueous lead stock solutions using a second-degree calibration curve according to DIN ISO 8466 (2004). Certified reference material (CRM) was analyzed for method validation and quality control. The method LOD and LOQ were 0.091 µg L^−1^ and 0.33 µg L^−1^, respectively.

Dissolved lead (*β*_Pb,diss_) was measured in filtered samples (0.45 µm), while total lead (*β*_Pb,tot_) was determined in unfiltered samples. For each subsample, dissolved and total lead were measured independently. All measurements were performed in duplicate, and mean values were used. Variability in total Pb among aliquots primarily reflects sample heterogeneity (e.g., uneven distribution of SPM) rather than analytical uncertainty. The magnitude of this variability was quantified using the coefficient of variation (CV). All measured values were included in the subsequent calculations; no data were excluded.

### Statistical analysis

The dissolved fraction of lead (*f*_d_) was calculated to evaluate the partitioning of lead between the dissolved and particulate phases in the river water samples. It was determined as the ratio of dissolved lead concentration (*β*_Pb,diss_) to total lead concentration (*β*_Pb,tot_):

$$f_{{\mathrm{d}}} = \frac{{\beta_{{\mathrm{Pb,diss}}} }}{{\beta_{{\mathrm{Pb,tot}}} }} \times 100{\text{ (\% )}}$$

Results are expressed in percent to facilitate interpretation. The parameter *f*_d_ was used in place of the conventional *K*_d_ to describe lead partitioning in natural river water containing SPM. As an operationally defined measure of the dissolved fraction, *f*_d_ provides a practical means of assessing lead mobility under field-relevant conditions, while differing conceptually from *K*_d_ values derived from solid–solution equilibrium models.

To evaluate how controlled EDTA spiking, differences between SP1 and SP2, and selected geochemical factors (such as pH and SPM content) influence lead partitioning in river water, two distinct statistical approaches were applied:

First, hypothesis testing was conducted using Welch’s *t*-tests, *F*-tests, and the Mann–Whitney *U* test to evaluate differences in parameters such as baseline lead and EDTA concentrations (*β*_Pb,diss_, *β*_Pb,tot_, *β*_EDTA_), *f*_d_, and the NEC between SP1 and SP2. Welch’s *t*-test was used to compare mean values because the datasets differ in sample size and, in some cases, exhibit unequal variances, as confirmed by *F*-tests reported in the Results section. Under these conditions, Welch’s test provides the most robust parametric option. Variance differences between sites were explicitly assessed with *F*-tests to quantify heteroscedasticity. In addition, the Mann–Whitney *U* test was applied to compare central tendencies based on rank distributions. This non-parametric approach was considered appropriate because environmental concentration data commonly deviate from normality and may include elevated values during flood events, which can disproportionately affect parametric tests.

Second, Bayesian modeling was used to determine the NEC for EDTA with R (R Core Team, 2023) and the bayesnec package (Fisher et al. 2024). The NEC model consists of a no-effect segment followed by a sigmoidal response curve, with the threshold estimated at the transition between these two sections. The Bayesian framework was chosen because it accommodates nonlinear concentration–response relationships and performs robustly with the heterogeneity and limited number of sampling dates characteristic of our environmental dataset, without requiring strict parametric assumptions.

Model fitting was conducted via Markov chain Monte Carlo (MCMC) sampling. Four chains with 15,000 iterations each (including 12,000 warm-up steps) were run. Default weakly informative priors provided by bayesnec were used. Convergence was assessed using standard diagnostics (*R̂* ≤ 1.01 and sufficient effective sample sizes). To evaluate robustness, each experiment was modeled six times, and the resulting NEC estimates were consistent to at least two significant figures. Posterior uncertainty was quantified using 95% Bayesian credible intervals (CrI, 2.5–97.5% posterior quantiles) for all model parameters, including the NEC. For visualization, posterior mean curves and their 95% CrI were plotted, and the NEC was shown together with its corresponding CrI derived from the NEC posterior distribution.

We applied both hypothesis testing and Bayesian dose–response modeling because this combination allowed us to address the heterogeneous characteristics of the dataset and to identify both site-specific differences and the EDTA concentrations at which changes in lead dynamics become detectable. Each approach has inherent limitations: hypothesis tests can be influenced by heteroscedasticity and small sample sizes, whereas Bayesian modeling relies on a predefined functional form and assumes independent observations. By applying both methods in parallel, we mitigated the limitations of each individual approach and obtained a coherent representation of central tendencies and associated uncertainty. This integrated strategy provides a robust and appropriate basis for evaluating EDTA-induced effects in this study.

### Lead speciation modelling

To mechanistically assess how EDTA affects lead partitioning under environmentally relevant conditions, pH-dependent Pb speciation was modeled using Visual MINTEQ (version 4.0.1). Dissolved Pb and EDTA concentrations were taken from our own measurements (median values for SP1 and SP2). Additional water-chemistry parameters required by the model (Ca^2+^, Mg^2+^, Na^+^, K^+^, Cl^−^, SO_4_^2−^, alkalinity, DOC) were obtained from the Lower Saxony Water Management, Coastal Defence and Nature Conservation Agency (NLWKN 2025). For consistency, we used monthly NLWKN monitoring data from May 2023 to June 2024, matching the period of our sampling campaign. The stations Heinde (upstream; analogue to SP1) and Sarstedt (downstream; analogue to SP2) were selected as the closest long-term monitoring sites on the Innerste River. Median values (12 monthly measurements per site) were used as model input.

Alkalinity (reported as acid capacity to pH 4.3) was converted to bicarbonate-equivalent concentrations prior to modeling. Measured DOC concentrations were used to represent natural organic matter (NOM). NOM complexation was modeled using the NICA–Donnan approach (Non-Ideal Competitive Adsorption combined with a Donnan electrostatic phase), which describes metal binding to heterogeneous organic matter. Simulations were performed over pH 4.0–10.0 in increments of 0.25 pH units. For interpretation, individual aqueous Pb species were grouped into chemically meaningful species groups:

- ΣPb–NOM: Pb–FA1 and Pb–FA2 complexes
- ΣPb–EDTA: PbEDTA^2−^, PbHEDTA^−^, PbH_2_EDTA(aq)
- ΣPb–free: Pb^2+^ and minor inorganic ion pairs
- ΣPb–carbonate: PbCO_3_(aq), Pb(CO_3_)_2_^2−^

In Visual MINTEQ, NOM is internally partitioned into two fulvic-acid subfractions (FA1, FA2), which represent binding-site classes rather than chemically discrete entities. Therefore, all FA1- and FA2-associated Pb complexes were summed and reported as ΣPb–NOM. Carbonate species were listed separately because they form the principal anionic Pb complexes under circumneutral freshwater conditions and show characteristic pH-dependent formation patterns. Minor inorganic ion pairs (e.g., PbHCO_3_^+^, PbCl^+^, PbSO_4_(aq)) and trace hydroxo species were grouped with ΣPb–free because they are generally considered weak, rapidly dissociating complexes in freshwater speciation models and do not constitute a distinct, diagnostically meaningful species group. To examine how increasing chelator availability shifts Pb partitioning, scenarios with elevated EDTA concentrations were also modeled.

Additional QA/QC information (including calibration data, LOD/LOQ values, recoveries, and representative chromatograms), diagnostic outputs from the statistical analyses (traceplots and prior–posterior comparisons), and all raw data (CSV file) are provided in the Supplementary Information. Detailed information related to the geochemical speciation modeling is also included, comprising the full input-parameter tables and an example output table showing Pb speciation across the entire pH range for one representative dataset.

## Results and discussion

### Initial studies

The first series of remobilization experiments (April 2022) used river water from SP1 (upstream) and applied EDTA spiking levels of 5–350 µg L^−1^ to investigate the lower concentration range in which EDTA-induced Pb remobilization has previously been reported (e.g., Jiann et al. 2013). The second series (May 2022) used river water from SP2 (downstream) and extended the spiking range to 1000 and 4000 µg L^−1^ to examine higher concentrations under downstream conditions influenced by WWTP effluent. In subsequent experiments, this extended EDTA range was applied at both sampling points to ensure comparability. Baseline EDTA concentrations (*β*_EDTA,0_) were measured prior to spiking and consistently remained below the minimum addition level of 5 µg L^−1^, confirming that all observed effects resulted from the added EDTA. Initial dissolved and total Pb concentrations (*β*_Pb,diss,0_ and *β*_Pb,tot,0_) were determined for both sites. At SP1, *β*_Pb,diss,0_ and *β*_Pb,tot,0_ were 1.3 µg L^−1^ and 7.8 µg L^−1^, respectively, corresponding to an initial *f*_d,0_ of 17%. In the second series at SP2, dissolved and total Pb concentrations were higher (2.3 µg L^−1^ and 16 µg L^−1^, respectively), yielding an *f*_d,0_ of 14%.

In the first series of remobilization experiments using SP1 river water, the dissolved fraction showed no systematic response to EDTA spiking in the range of 5–350 µg L^−1^. Instead, *f*_d_ fluctuated between 7 and 23% without a concentration-dependent pattern. These fluctuations were likely caused by insufficient homogenization of the bulk river water sample. Because aliquots were taken from a large volume containing native SPM, incomplete mixing may have resulted in uneven distribution of suspended particles during subsampling. The marked variation in total Pb across aliquots (6.8–18 µg L^−1^; 8.81 ± 3.07 µg L^−1^, CV = 35%) is consistent with this interpretation and indicates that particle heterogeneity contributed to the observed spread in *f*_d_. In subsequent experiments, this issue was mitigated through more thorough mixing before aliquoting.

**Fig. 4:**
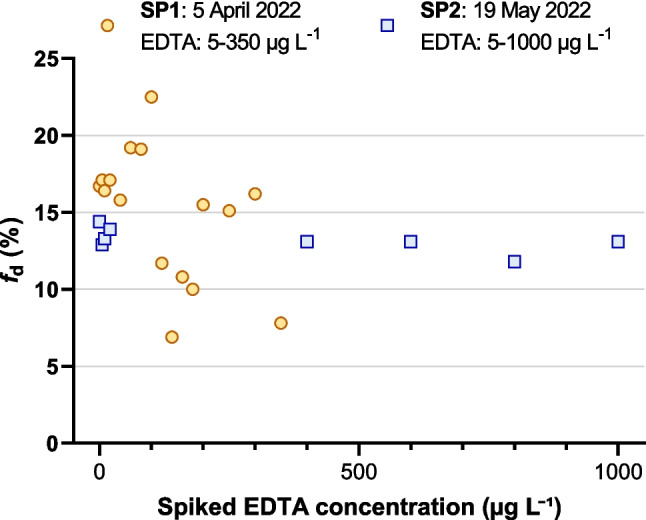
Dissolved lead fraction (*f*_d_) in response to EDTA spiking for two river water samples collected in spring 2022. SP1 (April 2022) showed *f*_d_ values of 7–23% at 5–350 µg L⁻^1^ EDTA, and SP2 (May 2022) showed *f*_d_ values of 12–14% at 5–1000 µg L⁻^1^. In both samples, *f*_d_ showed no concentration-dependent trend

The refined homogenization procedure markedly reduced variability in the second series of experiments. Using river water from SP2 and an extended EDTA spiking range of up to 1000 µg L^−1^, total Pb concentrations were consistent across aliquots (16.75 ± 0.89 µg L^−1^; CV = 5%), indicating that particle heterogeneity was effectively minimized. This consistency was also reflected in *f*_d_, which remained within a narrow range of 12–14%, even at the highest EDTA spiking levels. However, *f*_d_ showed no systematic increase with rising EDTA concentrations, and no NEC could be determined for this dataset. Figure 4 shows the *f*_d_ values obtained in the two initial series and their lack of a concentration-dependent response to EDTA spiking.

### Comprehensive analysis

To address the inconsistencies observed at lower EDTA concentrations, we expanded the experimental range to 0–4000 µg L^−1^. The first measurement in this series used water from SP1, located upstream of the largest WWTP on the Innerste, to minimize potential effluent influence. The initial EDTA concentration was 1.5 µg L^−1^, well below reported thresholds for measurable Pb remobilization. Initial Pb concentrations were 1.2 µg L^−1^ for *β*_Pb,diss_ and 8.1 µg L^−1^ for *β*_Pb,tot_, corresponding to an *f*_d_ of 15%. At 5 µg L^−1^ of EDTA spiking, an initial decrease in *f*_d_ to 6% was observed. Between 10 and 60 µg L^−1^, *f*_d_ remained near 7%, with slight increases to 8% at 100 and 200 µg L^−1^.

This initial decrease in *f*_d_ at low EDTA concentrations may be explained by two mechanisms and should be regarded as a working hypothesis. First, EDTA may bind to reactive surface sites on SPM, temporarily reducing dissolved Pb by forming ternary SPM–EDTA–Pb structures (Bradl 2004), which have been spectroscopically confirmed for other metal–EDTA systems on Fe-oxide surfaces (Norén and Persson 2010). Second, some of these surface sites may initially retain metals that form more stable EDTA complexes than Pb, such as Fe, Cu, or Ni (log *K*: Fe–EDTA 25.1; Cu–EDTA 18.8; Ni–EDTA 18.4; Pb–EDTA 18.0) (Burgess 2020). Complexation and release of these metals could allow dissolved Pb to sorb to the particles, producing the observed initial decrease in *f*_d_. As EDTA concentrations increase, free EDTA becomes available to desorb Pb from particle surfaces. This hypothesis is consistent with established EDTA speciation and metal–ligand exchange models in natural waters, where EDTA is predominantly present as metal–EDTA complexes and metal remobilization proceeds via metal–metal–EDTA exchange (Nowack 2002). Reported kinetics indicate that the rate and extent of Pb release depend on the competing metal–EDTA complex; for example, CaEDTA exchanges more rapidly than Fe(III)EDTA in homogeneous systems. Only limited Pb remobilization from hydrous ferric oxide occurs near pH 8, consistent with strong Pb sorption and surface-controlled kinetics under circumneutral conditions (Nowack et al. 2001).

In line with this working hypothesis, *f*_d_ began to increase at EDTA spiking levels of 600 µg L^−1^ and above, reaching 17% at concentrations beyond 1500 µg L^−1^. At these higher levels, the availability of free EDTA likely becomes sufficient to compete more effectively with particle-associated ligands, resulting in enhanced Pb release. Similar initial patterns were observed in some of the subsequent remobilization experiments, although not consistently across all datasets. The variability among experiments indicates that the response to EDTA spiking is governed by site-specific conditions, including the initial Pb speciation, the physicochemical properties of the SPM, pH, dissolved organic matter, and competing cations. Such dependencies are consistent with mechanistic work on aminopolycarboxylate chelators and with studies on sediment–water remobilization and trace-metal speciation in natural waters (Bordas and Bourg 1998; Jiann et al. 2013; Nowack 2002; Wojtkowska and Bogacki 2022).

**Fig. 5:**
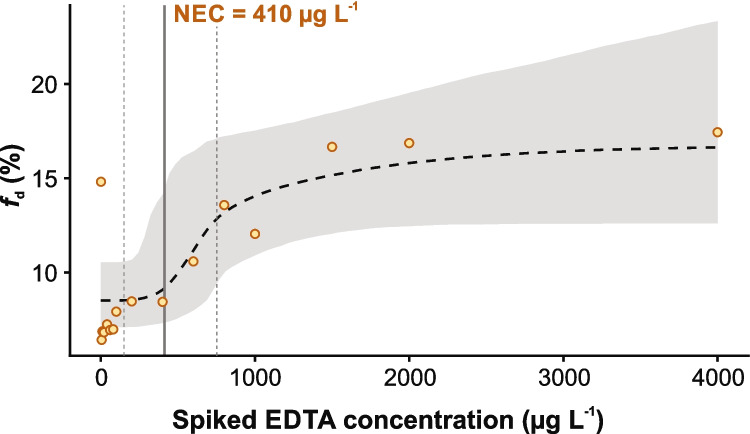
Dissolved lead fraction (*f*_d_) in response to EDTA spiking for a river water sample from SP1 (May 2023; *f*_d_ = 6–17%). The dashed line shows the posterior mean bayesnec fit with its 95% CrI (shaded). The vertical solid line marks the NEC estimate, and the flanking dashed lines show its 95% CrI (150–750 µg L^−1^)

Acknowledging this variability, we applied Bayesian concentration–response modeling to the extended dataset and obtained an NEC of 410 µg L^−1^ for EDTA-induced Pb remobilization in the SP1 sample (Fig. 5).

While the NEC was derived from batch experiments, the batch setup does not reproduce riverine hydrodynamics. Batch systems are nevertheless widely used to characterize geochemical interactions between dissolved ligands and particle-bound metals under controlled conditions, without the influence of shear stress, settling dynamics, or variable flow. Because native river water and in-situ SPM were used, the chemical composition of the Innerste River was preserved. The resulting NEC therefore reflects Pb–ligand interactions in the natural matrix, while recognizing that hydrodynamic effects were not part of the experimental design.

To examine the consistency of EDTA-induced Pb remobilization, we carried out an extended series of experiments in 2023 and 2024 at SP1 and SP2. These experiments, together with the assessment of baseline conditions at both sites, provide a more comprehensive dataset for evaluating Pb release under controlled batch conditions using native river water and in-situ SPM. The results are summarized in Table 1, which lists the baseline dissolved and total Pb concentrations (*β*_Pb,diss,0_ and *β*_Pb,tot,0_), the initial EDTA levels (*β*_EDTA,0_), the dissolved Pb fraction prior to EDTA spiking (*f*_d,0_), and the NEC values derived from the statistical analysis. While total Pb was predominantly measured in unfiltered, HNO_3_-acidified subsamples, aqua regia digestion was applied in selected experiments to assess potential effects of digestion on NEC estimation; the corresponding entries are marked “AR”.

**Table 1:** Baseline Pb concentrations (*β*_Pb,diss,0_ and *β*_Pb,tot,0_), the dissolved Pb fraction before EDTA spiking (*f*_d,0_), initial EDTA levels (*β*_EDTA,0_), and NEC values from remobilization experiments conducted in 2023 and 2024. Total Pb values refer to unfiltered, HNO_3_-acidified samples; entries marked “AR” denote measurements after aqua regia digestion

Measurement date	Sampling location	*β*_Pb,diss,0_ (µg L^−1^)	*β*_Pb,tot,0_ (µg L^−1^)	*f*_d,0_ (%)	*β*_EDTA,0_ (µg L^−1^)	Shaking time	NEC (µg L^−1^)
2023-05-31	SP1	1.2	8.1	15	1.5	2 h	410
2023-07-17	SP1	1.8	15	12	0.77	2 h	370
2023-08-30	SP1	1.5	16	9	0.80	2 h	420
2023-09-26	SP1	0.49	11	4	3.3	2 h	280
2023-10-18	SP2	0.64	7.1	9	1.9	2 h	230
2024-02-19	SP1	0.61	77	1	3.1	2 h	300
	SP1	0.41	72	1	3.1	24 h	290
2024-03-04	SP1	1.0	17	6	2.0	2 h	320
	SP1	1.1	16	7	2.0	2 h	310
2024-03-11	SP1	1.1	15	7	0.89	2 h	230
	SP1	1.8	29	6	0.89	24 h	480
2024-04-02	SP1	0.20	25	1	0.68	2 h	530
	SP1	0.076	80	< 1	0.68	24 h	380
2024-04-03	SP1	0.39	11	4	1.2	2 h	270
	SP1	0.39	20 (AR)	2	1.2	2 h	290
	SP1	0.64	7.2	9	1.2	24 h	280
	SP1	0.64	9.1 (AR)	7	1.2	24 h	290
2024-04-23	SP2	0.71	7.9	9	2.2	2 h	220
2024-05-14	SP1	0.54	7.2	8	1.6	2 h	260
	SP2	0.50	7.9	6	3.1	2 h	210
2024-06-05	SP1	0.76	11	7	1.6	2 h	290
	SP2	0.75	10	8	3.8	2 h	320
2024-06-11	SP1	0.86	13	7	1.8	2 h	290
	SP2	0.69	15	5	2.9	2 h	440
2024-06-19	SP1	0.11	10	1	1.5	2 h	300

All measured and calculated concentration values have been rounded to two significant figures. In order to enhance readability, the percentage values are rounded to the nearest whole number

### Baseline parameters

**Fig. 6:**
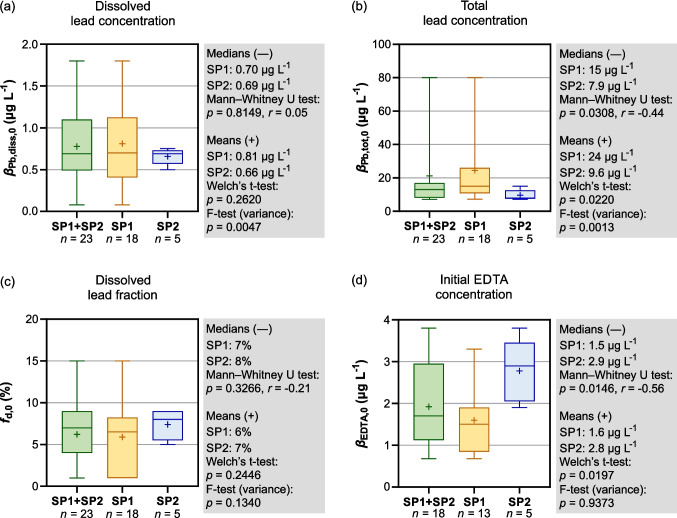
Comparison of baseline conditions at SP1 (upstream), SP2 (downstream), and the combined dataset (SP1 + SP2) for **a** dissolved Pb, **b** total Pb, **c** dissolved Pb fraction, and **d** initial EDTA concentration. Group differences were assessed using Mann–Whitney *U* tests, Welch’s *t*-tests, and *F*-tests (*p* < 0.05). Effect sizes (*r*) are reported according to Cohen’s benchmarks

Across the combined dataset from SP1 and SP2, *β*_Pb,diss,0_ ranged from 0.08 to 1.8 µg L^−1^, with a median of 0.69 µg L^−1^ and a mean of 0.78 µg L^−1^ (Fig. 6a). These concentrations are consistent with the legacy of Pb contamination associated with historical mining and metallurgical activities in the Harz Mountains. The average dissolved Pb concentration approaches the AA-EQS of 1.2 µg L^−1^, indicating that additional Pb release induced by anthropogenic chelating agents could be environmentally relevant in this system.

For SP1 and SP2, the medians (0.70 vs. 0.69 µg L^−1^) and means (0.69 vs. 0.66 µg L^−1^) of *β*_Pb,diss,0_ were nearly identical. To assess site-specific differences, we applied Mann–Whitney *U* tests (adjusted for ties), Welch’s *t*-tests, and *F*-tests. The Mann–Whitney *U* test showed no significant difference in rank distributions (*p* = 0.8149, *r* = 0.05), and Welch’s *t*-test indicated no significant difference in mean values (*p* = 0.2620). The *F*-test, however, indicated a significant difference in variance between sites (*p* = 0.0047). Overall, these results show that dissolved Pb concentrations did not differ detectably between upstream and downstream conditions in our dataset.

With respect to total Pb, values ranged from 7.1 to 80 µg L^−1^ (median 13 µg L^−1^; mean of 21 µg L^−1^) across both sites. During the observation period, a flood on 19 February 2024 was associated with particularly elevated total Pb concentrations (> 70 µg L^−1^). Similar increases during high-flow conditions have been reported for the Litavka River in the Czech Republic, where floodwaters mobilized historically contaminated sediments and enhanced downstream metal transport (Žák et al. 2009). A similar pattern is plausible for the Innerste, where legacy mining contamination remains a potential source of particle-bound Pb during high-flow events.

Site-specific differences were more pronounced for total Pb. At SP1, *β*_Pb,tot,0_ showed a median of 15 µg L^−1^ and a mean of 24 µg L^−1^, whereas both metrics were lower at SP2 (median 7.9 µg L^−1^; mean 9.6 µg L^−1^). The Mann–Whitney *U* test indicated a significant difference in rank distributions (*p* = 0.0308, *r* = −0.44), corresponding to a medium effect size according to Cohen’s benchmarks. This was supported by significant differences in mean concentrations (Welch’s *t*-test, *p* = 0.0220) and by a significant variance difference (*F*-test, *p* = 0.0013) (Fig. 6b). The significant decrease in total Pb from SP1 to SP2 cannot be explained by dilution alone and is consistent with a combination of dilution and particle-related processes along the flow path. Dissolved Pb, however, remained nearly unchanged between the two sites, indicating that downstream conditions did not lead to a corresponding reduction in the dissolved fraction.

To assess whether downstream conditions influenced Pb partitioning, we examined the dissolved fraction, *f*_d_. The median *f*_d_ across both sites was 7%, with an overall mean of 6% (Fig. 6c). At SP2, *f*_d_ values showed a median of 8% and a mean of 7%, whereas SP1 exhibited slightly lower values (median 7%; mean 6%). These differences were not statistically significant. The Mann–Whitney *U* test (*p* = 0.3266, *r* = −0.21), Welch’s *t*-test (*p* = 0.2446), and the *F*-test (*p* = 0.1340) all indicated no significant differences in ranks, means, or variances between sites. Thus, the dataset does not provide evidence that the wastewater treatment plant affected Pb partitioning.

Differences in baseline Pb concentrations and *f*_d_ values between SP1 and SP2 are likely attributable to temporal variability in river conditions, including fluctuations in flow velocity, changes in suspended particle composition, and localized sediment–water interactions. Comparable influences have been reported in coastal systems. For example, Liu et al. (2023) showed that hydrodynamic conditions and sediment characteristics can shape the distribution and remobilization of heavy metals in coastal waters off the Shandong Peninsula (China). Although our study focuses on a riverine system, similar hydrodynamic and particle-associated factors may contribute to the spatial variability observed in the Innerste.

In addition to these factors, variations in pH could also be relevant. Across the measured pH range (7.1–8.8), however, no significant correlation with *f*_d,0_ was observed (Spearman *ρ* = −0.02, *p* = 0.96, *n* = 13; see Fig. S3-1). SPM concentrations ranged from 5.1 to 69 mg L^−1^ and were particularly elevated during flood events. A moderate negative trend with *f*_d,0_ was observed (*ρ* = −0.54, *p* = 0.06), but this pattern was driven entirely by three high-SPM observations (> 30 mg L^−1^). When these data points were excluded, the correlation disappeared (*ρ* = −0.02, *p* = 0.95, *n* = 10; see Fig. S3-2), indicating that the apparent trend does not represent a consistent relationship. No significant correlations with other parameters, including total Pb, were identified. Additional factors such as SPM composition or specific Pb-binding phases may influence Pb partitioning and remain potential directions for future work.

In contrast to the behavior of Pb, EDTA showed a clear downstream increase. Across all samples, EDTA concentrations averaged 1.9 µg L^−1^ with a median of 1.7 µg L^−1^ (range: 0.68–3.8 µg L^−1^). At SP1, EDTA concentrations had a mean of 1.6 µg L^−1^ and a median of 1.5 µg L^−1^, whereas at SP2 both metrics were considerably higher (mean 2.8 µg L^−1^; median 2.9 µg L^−1^; Fig. 6d). The Mann–Whitney *U* test indicated a significant difference in rank distributions (*p* = 0.0146, *r* = −0.56), and Welch’s *t*-test showed a significant difference in means (*p* = 0.0197). In contrast, the *F*-test showed no difference in variances between sites (*p* = 0.9373). Overall, these results indicate that the WWTP substantially increases EDTA concentrations between SP1 and SP2.

### NECs derived from the EDTA remobilization experiments

**Fig. 7:**
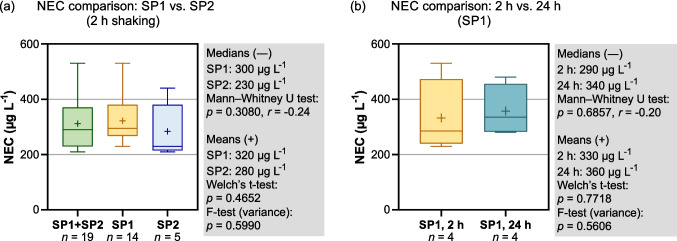
**a** Comparison of NEC values for EDTA at SP1 (upstream), SP2 (downstream), and the combined dataset (SP1 + SP2) for a shaking duration of 2 h. **b** Comparison of NEC values at SP1 for shaking durations of 2 h and 24 h. Statistical tests follow the procedures described in Fig. 6

We next evaluated NECs for EDTA as derived from the Bayesian dose–response model applied to the remobilization experiments. At the standard shaking duration of 2 h, NECs across all experiments (SP1: *n* = 14; SP2: *n* = 5) ranged from 210 to 530 µg L^−1^, with a median of 290 µg L^−1^ and a mean of 310 µg L^−1^. At SP1, NEC values showed a median of 300 µg L^−1^ and a mean of 320 µg L^−1^, whereas at SP2 they were slightly lower (median 230 µg L^−1^; mean 280 µg L^−1^) but within a comparable range. Statistical testing indicated no significant site-specific differences: the Mann–Whitney *U* test showed no difference in rank distributions (*p* = 0.3080), and Welch’s *t*-test showed no difference in means (*p* = 0.4652) (Fig. 7a). Across both sites, the NECs are roughly two orders of magnitude higher than EDTA concentrations measured in the Innerste, indicating that ambient EDTA levels are far below those required to measurably remobilize Pb from SPM under the examined conditions.

To assess whether shaking duration affects NEC estimation, NEC values from four water samples at SP1 were compared between the 2-h and 24-h experiments. Medians and means were similar (290 vs. 340 µg L^−1^ and 330 vs. 360 µg L^−1^, respectively). Statistical testing indicated no significant differences: the Mann–Whitney *U* test showed no difference in rank distributions (*p* = 0.6857), and Welch’s *t*-test indicated no difference in means (*p* = 0.7718) (Fig. 7b). These results indicate that extending the shaking duration from 2 to 24 h does not substantially affect the NEC, suggesting that shorter shaking times are sufficient for estimating the EDTA concentration required to remobilize Pb from SPM under the tested conditions. Although the NEC values did not differ statistically between shaking durations, a consistent pattern emerged in the increase of the dissolved Pb fraction. We quantified this as the absolute increase (Δ*f*_d_) between the initial and maximum *f*_d_:

$$\triangle f_{d}=f_{d, \mathrm{max}}-f_{d,0}$$

**Fig. 8:**
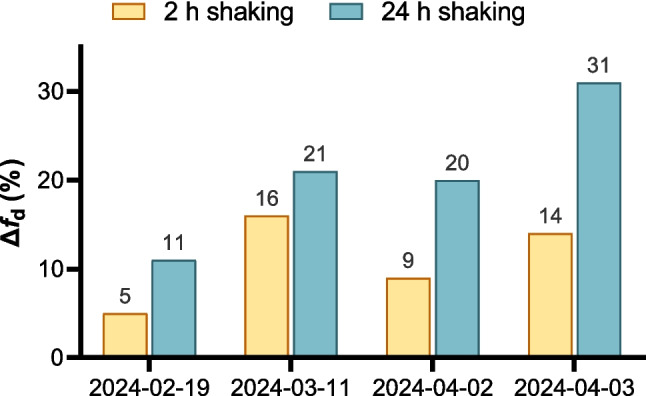
Absolute increase in dissolved lead fraction (Δ*f*_d_) in Innerste river water induced by EDTA spiking for four sampling dates at SP1 in 2024. For each date, Δ*f*_d_ was calculated as the difference between the initial dissolved fraction (*f*_d,0_) and the maximum *f*_d_ observed within the spiking series. Bars compare 2-h and 24-h shaking

Across all sampling dates, the 24-h shaking period resulted in a larger Δ*f*_d_ than the 2-h period. In the 19 February 2024 sample, Δ*f*_d_ increased from 5% (1% → 6%) after 2 h to 11% after 24 h. In the 11 March sample, the increase was 16% vs. 21%, and in the 2 April sample, 9% vs. 20%. The most pronounced difference was observed in the 3 April 2024 sample, where Δ*f*d increased from 14 to 31% (Fig. 8).

These observations indicate that longer shaking durations increase the extent of Pb remobilization from SPM. However, despite the differences in Δ*f*_d_, the NECs remained statistically indistinguishable between the 2-h and 24-h experiments. This is relevant because the 2-h duration was selected based on literature showing that Pb–EDTA interactions typically approach near-equilibrium within the first 3 h (Parsadoust et al. 2022). Thus, a 2-h shaking period appears sufficient for NEC estimation, whereas the 24-h experiments primarily capture the additional remobilization that can occur under prolonged particle–chelator contact without shifting the NEC threshold.

**Fig. 9:**
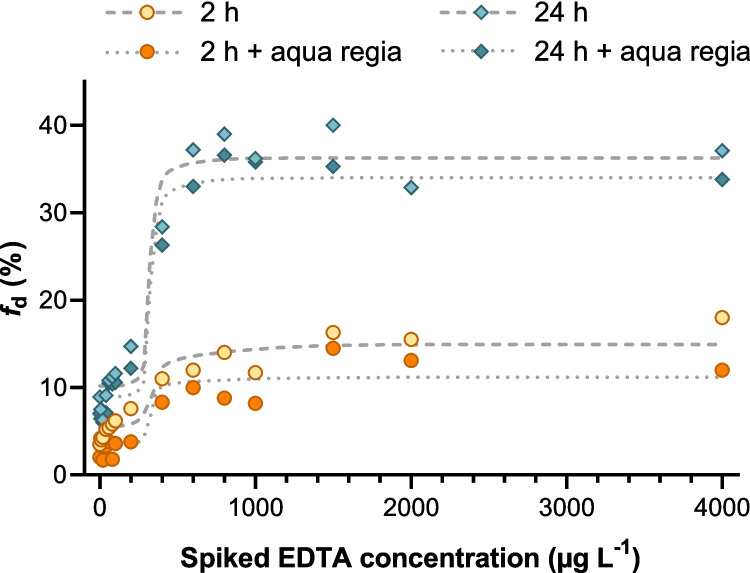
Dissolved lead fraction *f*_d_ as a function of spiked EDTA concentration for a river water sample from SP1 collected on 3 April 2024. Four batch treatments were tested (2 h, 2 h + aqua regia, 24 h, 24 h + aqua regia). Light-filled markers indicate samples without aqua regia; dark-filled markers indicate aqua regia treatment. Gray dashed lines show the posterior mean model fits for the treatments without aqua regia, and gray dotted lines show the corresponding fits for the treatments with aqua regia (Credible intervals omitted for clarity)

Building on the evaluation of shaking duration, we also evaluated whether aqua regia digestion affects NEC estimation. For the SP1 sample collected on 3 April 2024, we examined how microwave digestion of the aliquots used for determining total Pb influenced the resulting *f*_d_ values and, consequently, the NEC. Unfiltered aliquots were spiked with EDTA, subjected to aqua regia microwave digestion, and analyzed for total Pb. As expected, digestion increased the measured total Pb concentrations, which in turn lowered the corresponding *f*_d_ values at each EDTA spiking level (Fig. 9).

Despite the shift in total Pb and *f*_d_ values caused by digestion, the resulting NECs changed only slightly. For the 2-h shaking duration, the NEC was 270 µg L^−1^ without digestion and 290 µg L^−1^ with digestion. For the 24-h experiments, the corresponding values were 280 µg L^−1^ and 290 µg L^−1^. These small differences do not indicate a systematic effect of digestion on the NEC. Given this close agreement, and considering that extended shaking also did not alter the NEC, microwave digestion with aqua regia provides no methodological advantage. The simpler procedure without digestion and with the shorter contact time appears to be sufficient and practical for determining NECs when SPM is the solid phase under investigation.

Given this close agreement, and considering that longer shaking durations also did not affect the NEC, microwave digestion with aqua regia does not provide a methodological advantage. Thus, the simpler procedure without digestion and with a shorter contact time appears sufficient and practical for determining NECs related to Pb remobilization from SPM in river water.

After evaluating method-related parameters, we examined total Pb variability across the full dataset. Building on the CV analysis from the initial experiments, we quantified CVs for all remaining datasets to characterize the reproducibility of total Pb measurements under differing SPM conditions.

Because total Pb was measured separately for each EDTA-spiked aliquot, incomplete homogenization of the bulk sample, particularly under high-SPM conditions, can lead to differences in total Pb between aliquots. Such variability affects the calculated *f*_d_ values and could, in principle, introduce uncertainty into NEC estimation. To assess the magnitude of this effect, we quantified total Pb variability for all experiments. Most datasets showed low to moderate variability (21 out of 25 datasets with CV values between 1 and 11%), indicating that aliquoting was generally reproducible.

Four datasets, however, exhibited elevated variability (CVs of 16%, 30%, 34%, and 54%). The three highest CV values (30%, 34%, and 54%) occurred in experiments with 24-h shaking or aqua regia digestion, and all coincided with moderate to high SPM loads (18–69 mg L^−1^), where sedimentation and particle aggregation make aliquoting more sensitive to small differences in mixing and timing. One moderately elevated CV (16%) provides an illustrative example of operator-dependent effects: on 4 March 2024, two experimenters processed the same water sample independently, yielding CVs of 16% and 11%, respectively. This comparison suggests that aliquoting precision can vary between operators, even under otherwise identical conditions.

Paired experiments performed with the same water sample but different shaking durations demonstrated that increased variability did not cause a consistent directional shift in NEC. For example, on 11 March 2024, the NEC increased from 230 µg L^−1^ (2 h, CV = 8%) to 480 µg L^−1^ (24 h, CV = 34%), whereas on 2 April 2024, it decreased from 530 µg L^−1^ (2 h, CV = 11%) to 380 µg L^−1^ (24 h, CV = 54%). Thus, higher variability in total Pb introduces random uncertainty rather than systematic bias, and all NEC values remained within the same order of magnitude.

To contextualize the NEC values determined for the Innerste (210–530 µg L^−1^), we compared them with studies that examined EDTA–metal interactions in river water. Jiann et al. (2013) analysed dissolved and particulate metals in eight Texas rivers and conducted a principal component analysis that linked EDTA concentrations (≈40–500 nM; 12–146 µg L^−1^) with elevated dissolved trace-metal levels. EDTA, together with DOC, pH, and SPM, was among the dominant factors controlling metal partitioning, exerting a strong influence on dissolved Cd and Zn and a weaker influence on Cu, Ni, and Pb. Although no concentration–response relationship was established, these findings show that EDTA-related changes in metal partitioning can occur at comparatively low EDTA concentrations in DOC-rich and urban-influenced river systems.

Gonsior et al. (1997) investigated river-sediment suspensions at much higher solid-to-liquid ratios and reported a no-observed-effect level (NOEL) for Pb of 8 µM EDTA (≈2340 µg L^−1^). This higher NOEL reflects the substantial buffering capacity of slurry-type sediment loads, whereas the NECs determined in the present study are based on controlled concentration–response experiments in native river water with environmentally realistic SPM concentrations.

Batch studies of EDTA and related chelants have shown desorption “edges” over restricted concentration intervals (e.g., Bordas and Bourg 1998), indicating that threshold-like behavior depends on solid–solution ratios and geochemical conditions. Taken together, these comparisons show that Pb remobilization by EDTA is strongly influenced by matrix characteristics, organic ligands, and particle loading.

### Environmental implications

Ambient EDTA concentrations measured in the Innerste (0.8–3.8 µg L^−1^) are more than two orders of magnitude below the NEC range determined in this study. Although EDTA can, depending on geochemical conditions, either reduce metal toxicity or modify metal speciation, available bioassay data indicate that metal–EDTA complexes generally exhibit substantially lower toxicity than their free metal ions. Sorvari and Sillanpää (1996) reported marked toxicity reductions for several metal–EDTA complexes in Daphnia magna, and Microtox assays showed comparatively low toxicity of Pb–EDTA (Sillanpää and Oikari 1996). Together with the low intrinsic toxicity of EDTA at environmentally relevant levels, these findings indicate that present EDTA concentrations in the Innerste do not raise ecotoxicological concern with respect to Pb mobilization.

EDTA is persistent in surface waters because only Fe(III)–EDTA undergoes appreciable photodegradation, with rates depending on the water matrix and light regime (Kari and Giger 1995; Metsärinne et al. 2001). At the concentrations observed in the Innerste, this persistence primarily results in downstream transport rather than changes in short-term Pb speciation.

From an environmental-management perspective, the NEC values establish a quantitative threshold above which EDTA begins to influence Pb partitioning in the Innerste. Because ambient EDTA levels remain far below this threshold, chelate-induced mobilization of legacy Pb is unlikely under present conditions. The NEC therefore provides a practical reference for evaluating whether changes in EDTA inputs, e.g., from WWTP, could alter Pb mobility in contaminated river systems.

### Geochemical Pb speciation modeling and mechanistic interpretation

To provide a mechanistic interpretation of the experimentally derived NEC values, we modeled pH-dependent dissolved Pb speciation using median Pb and EDTA concentrations measured in this study together with site-specific water-chemistry parameters obtained from the NLWKN state monitoring program. NOM was represented in the model by the dissolved fraction quantified as DOC from the state monitoring program. Background speciation was simulated separately for SP1 and SP2. Because background EDTA and Pb concentrations, as well as the other site-specific water-chemistry parameters, differed only slightly between SP1 and SP2, the resulting baseline speciation patterns were nearly identical. For this reason, the speciation is discussed for SP2, which has the slightly higher background EDTA concentration and therefore represents the more conservative case for evaluating EDTA–Pb competition.

**Fig. 10:**
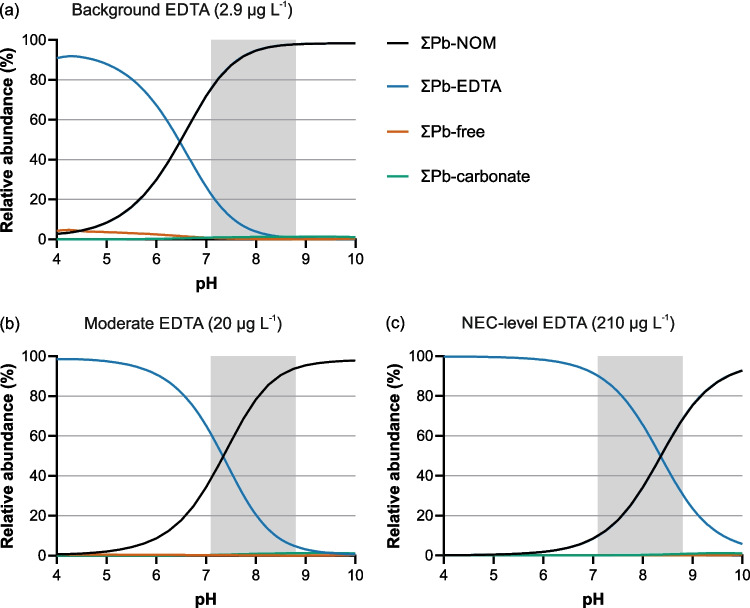
Modeled pH-dependent relative abundance of dissolved Pb species at SP2 for **a** background EDTA (2.9 µg L^−1^), **b** moderately elevated EDTA (20 µg L^−1^), and **c** NEC-level EDTA (210 µg L^−1^). Species groups include ΣPb–NOM, ΣPb–EDTA, ΣPb–carbonate, and ΣPb–free. The shaded region indicates the pH interval measured in the Innerste River (7.1–8.8). Modeling was performed using site-specific water-chemistry data and DOC-based representation of dissolved NOM

At background EDTA concentrations for SP2 (median 2.9 µg L^−1^) and within the pH range measured in the Innerste (7.1–8.8), the simulations predict that dissolved Pb is present predominantly as ΣPb–NOM, with ΣPb–EDTA contributing only minimally. Across this pH interval, ΣPb–NOM accounts for roughly 75–98% of dissolved Pb, whereas ΣPb–EDTA represents about 22% at the lower end of the pH range and decreases to < 1% toward higher pH (Fig. 10a).

When the EDTA concentration in the model is increased to 20 µg L^−1^, the speciation model predicts that ΣPb–NOM remains the predominant species across most of the pH range relevant for the Innerste, accounting for approximately 37–94% of dissolved Pb. ΣPb–EDTA increases to about 62% at the lower end of this interval but declines to < 10% toward higher pH (Fig. 10b). Although the model indicates a partial shift in speciation at this concentration, this does not correspond to a measurable change in *f*_d_ in the batch experiments, in which low to moderate EDTA additions did not produce an observable response.

At an EDTA concentration corresponding to the lower boundary of the NEC range (210 µg L^−1^), the model predicts a marked shift in speciation within the pH range relevant for the Innerste. ΣPb–EDTA reaches approximately 90% of dissolved Pb at the lower end of this interval and decreases to about 31% toward higher pH, while ΣPb–NOM increases from roughly 9% to 68% (Fig. 10c). Over most of the measured pH range, ΣPb–EDTA thus accounts for the largest single fraction of dissolved Pb, before ΣPb–NOM becomes dominant toward the upper end of the pH interval. This modeled shift in the balance between NOM-bound and EDTA-bound Pb occurs at EDTA concentrations comparable to those that produced the threshold-like rise in *f*_d_ in the concentration–response experiments.

Taken together, the NEC experiments and the speciation modeling provide complementary insights into Pb–ligand interactions in the Innerste. The NEC experiments establish the EDTA concentrations at which measurable increases in the dissolved Pb fraction occur, but they do not identify which other ligands control Pb partitioning. The speciation modeling offers the mechanistic interpretation for these thresholds by showing that, under present water-chemistry conditions, dissolved NOM is predicted to dominate Pb complexation at ambient EDTA levels. In combination, these approaches indicate that EDTA concentrations would need to exceed current levels by more than two orders of magnitude before substantially altering dissolved Pb.

## Conclusion

This study quantified the EDTA concentration required to remobilize Pb from SPM in the Innerste River and found that experimentally derived NEC values (210–530 µg L^−1^) are far above ambient EDTA levels. Placed in a broader environmental context, these NECs lie within the range of concentrations reported to influence metal partitioning in other aquatic systems, while remaining well separated from the much lower EDTA levels observed in the Innerste. The NECs were robust across methodological variations, including shaking duration and the use or absence of aqua regia digestion. Geochemical speciation modeling further showed that Pb remains NOM-bound under current EDTA levels and shifts toward Pb–EDTA formation only at concentrations near the NEC.

Although Pb–EDTA interactions can vary with site-specific properties such as pH, DOC, competing cations, and SPM composition, present EDTA levels in the Innerste are too low to alter Pb partitioning. The NEC therefore provides a practical reference for assessing whether future changes in EDTA inputs, such as those associated with wastewater effluent, could influence Pb mobility in contaminated river systems. Further work on SPM composition and geochemical controls would help extend this approach to other riverine settings.

## Supplementary Information

Below is the link to the electronic supplementary material.

MOESM1 (DOCX 2.71 MB)

MOESM2 (CSV 13.2 KB)

## Data Availability

The data supporting the findings of this study are available within the paper and its Supplementary Information files. Any additional raw data files required in another format can be obtained from the corresponding author upon request.
