# Supplementary material for: EDTA-induced remobilization of lead from suspended particulate matter in contaminated water samples from the Innerste River: a statistical evaluation
Source: Environ Sci Pollut Res Int. 2026 Feb 7;33(8):3220–36. doi: 10.1007/s11356-026-37480-x (PMC13005870; doi:10.1007/s11356-026-37480-x)
Supplement: Supplementary file 1 — (DOCX 2.71 MB) [file 11356_2026_37480_MOESM1_ESM.docx]

**Supplementary Information**

**EDTA-induced remobilization of lead from suspended particulate matter in contaminated water samples from the Innerste River: a statistical evaluation**

Jan Klaus Hinrichs*, Markus Herrmann, Aaron Bauer, Dieter Steffen

Department of Chemistry, Institute of Biology and Chemistry, University of Hildesheim, Universitätsplatz 1, 31141 Hildesheim, Germany

*Corresponding author: jan.hinrichs@uni-hildesheim.de

**S1. GC-NPD analysis of EDTA:** Example Chromatograms, Calibration, QA/QC

**Figure S1-1**. Representative GC-NPD chromatogram of a calibration standard containing EDTA (4 µg L⁻¹) and the internal standard 1,2-PDTA (20 µg L⁻¹) after derivatization to the corresponding *n*-propyl esters
according to DIN EN ISO 16588:2004-02. The two annotated peaks correspond to the
EDTA-*n*-propyl ester (Peak 1) and the 1,2-PDTA-*n*-propyl ester (Peak 2).

**Figure S1-2**. Representative blank chromatogram. No EDTA *n*-propyl ester peak was detected; only the internal standard (1,2-PDTA *n*-propyl ester) appears.

**Figure S1-3**. Representative chromatogram of a river water sample from SP2 (2024-06-05) after sample preparation and derivatization.

**Table S1-1**. Representative calibration data for the GC–NPD determination of EDTA: peak areas of EDTA and the internal standard (1,2-PDTA) and their area ratios.

| ***β*_EDTA_ (µg L⁻¹)** | ***A*_EDTA_** | ***β*_1,2-PDTA_ (µg L⁻¹)** | ***A*_1,2-PDTA_** | ***A*_EDTA_/*A*_1,2-PDTA_** |
| --- | --- | --- | --- | --- |
| 0.50 | 23989 | 20.0 | 575240 | 0.0417 |
| 1.0 | 33890 | 20.0 | 583839 | 0.0580 |
| 2.0 | 82755 | 20.0 | 669145 | 0.1237 |
| 4.0 | 131001 | 20.0 | 566995 | 0.2310 |
| 6.0 | 187132 | 20.0 | 544323 | 0.3438 |
| 8.0 | 258688 | 20.0 | 566189 | 0.4569 |
| 10.0 | 302227 | 20.0 | 537496 | 0.5623 |

The limits of detection (LOD) and quantification (LOQ) were calculated according to DIN 32645 using a significance level of *α* = 5%.

**Table S1-2**. Summary of calibration and statistical parameters for the GC-NPD determination of EDTA
(DIN 32645, *α* = 5%).

| Parameter | Symbol | Value |
| --- | --- | --- |
| Slope of calibration curve | *b* | 0.0555 |
| Intercept of calibration curve | *a* | 0.0099 |
| Coefficient of determination | *R*² | 0.9996 |
| Residual standard deviation (standard error) | *s*ᵧ | 0.0044 |
| Method standard deviation | *s*ₓ₀ | 0.0787 |
| Relative method standard deviation | *v*ₓ₀ | 1.75% |
| Limit of detection (α = 5%) | LOD | 0.19 µg L⁻¹ |
| Limit of quantification (α = 5%) | LOQ | 0.70 µg L⁻¹ |

**Figure S1-4**. EDTA calibration curve (0.5-10 µg L⁻¹) including the 95% confidence interval (red) and the 95% prediction interval (blue) according to DIN 32645.

**S2. GF-AAS analysis of Pb:** Calibration, QA/QC, and Example Data

**Table S2-1**. Representative calibration data for the GF-AAS determination of Pb in the low (0.125-10 µg L⁻¹, left) and high (2-10 µg L⁻¹, right) working ranges are shown. Both calibration sets include a blank measurement (0 µg L⁻¹). Calibration standard concentrations (*β*_Pb_) and the corresponding integrated instrument signals (Ext.) are listed.

| ***β*_Pb_ (µg L⁻¹)** | ***Ext.*** |  | ***β*_Pb_ (µg L⁻¹)** | ***Ext.*** |
| --- | --- | --- | --- | --- |
| 0 | -0.0006 |  | 0 | 0.00122 |
| 0.125 | 0.00778 |  | 2.00 | 0.01668 |
| 0.250 | 0.01389 |  | 5.00 | 0.03571 |
| 0.500 | 0.02551 |  | 10.0 | 0.0674 |
| 1.00 | 0.04839 |  | 15.0 | 0.09982 |
| 2.00 | 0.09495 |  | 20.0 | 0.12672 |
| 4.00 | 0.18400 |  |  |  |
| 6.00 | 0.26533 |  |  |  |
| 8.00 | 0.34990 |  |  |  |
| 10.0 | 0.41156 |  |  |  |

**Table S2-2**. Summary of calibration and statistical parameters for the GF-AAS determination of Pb in the low (0.125-10 µg L⁻¹) and high (2-10 µg L⁻¹) working ranges (DIN ISO 8466-2, *α* = 5%).

| Parameter | Symbol | Value for  low range  (0.125-10 µg L⁻¹) | Value for  high range  (2-10 µg L⁻) |
| --- | --- | --- | --- |
| Calibration function |  | *y* = *c*·*x*² + *b*·*x* + *a* | *y* = *c*·*x*² + *b*·*x* + *a* |
| Quadratic coefficient | *c* | -7.895 × 10⁻⁴ | -3.966 × 10⁻^5^ |
| Linear coefficient | *b* | 4.927 × 10⁻² | 7.063 × 10⁻^3^ |
| Constant coefficient | *a* | 5.075 × 10⁻⁴ | 1.697 × 10⁻^3^ |
| Coefficient of determination | *R*² | 0.9998 | 0.9997 |
| Residual standard deviation | *s*ᵧ | 2.73 × 10⁻³ | 1.10 × 10⁻^3^ |
| Method standard deviation | *s*ₓ₀ | 6.18 × 10⁻² | 1.73 × 10⁻^1^ |
| Relative method standard deviation | *v*ₓ₀ | 1.94% | 1.99% |
| Limit of detection | LOD | 0.09 µg L^‑1^ | 0.38 µg L^‑1^ |
| Limit of quantification | LOQ | 0.33 µg L^‑1^ | 1.40 µg L^‑1^ |

**Certified reference material (CRM)**

As an external quality control, the certified reference material *Trace Metals 1-WP* (Sigma-Aldrich, lot LRAC9388) was analyzed as a 1:100 dilution. According to the certificate, the Pb concentration is 1079 ± 10 µg/L. Across *n* = 3 replicate measurements, we obtained a mean value of 1007 ± 58 µg/L, corresponding to a recovery of 93%. This confirms that the calibration and measurement procedure provided accurate results within the expected uncertainty range.

**S3. Relationships between dissolved Pb fraction (*f*_d,0_) and selected parameters**

**Figure S3-1**. Left: Scatter plot of pH versus the dissolved Pb fraction (*f*_d,0_) for all measurements where both parameters were available. Across the investigated pH range (7.1-8.7), no significant correlation was found (Spearman *ρ* = -0.02, *p* = 0.96, *n* = 13).

Right: Scatter plot of total Pb concentration (*β*_Pb,tot,0_) versus *f*_d,0_ for all measurements with available data. A moderate negative trend is visually apparent, but it was not statistically significant (Spearman *ρ* = −0.41, *p* = 0.0848, *n* = 19). The highest total Pb values drive most of the apparent pattern; after excluding these high-Pb observations, the association disappears (not shown).

**Figure S3-2**. Left: Scatter plot of suspended particulate matter (SPM) versus dissolved Pb fraction (*f*_d,0_) for all measurements with available SPM data (SPM = 5.1-69 mg L⁻¹). A moderate negative trend is visible, but it is not statistically significant (Spearman *ρ* = −0.54, *p* = 0.06, *n* = 13).

Right: Same dataset excluding the three high-SPM observations (> 30 mg L⁻¹), resulting in an SPM range of 5.1-17.7 mg L⁻¹. After removing these observations, no association remained (*ρ* = −0.02, *p* = 0.95, *n* = 10).

**S4. Bayesian NEC model computation (*bayesnec*)**


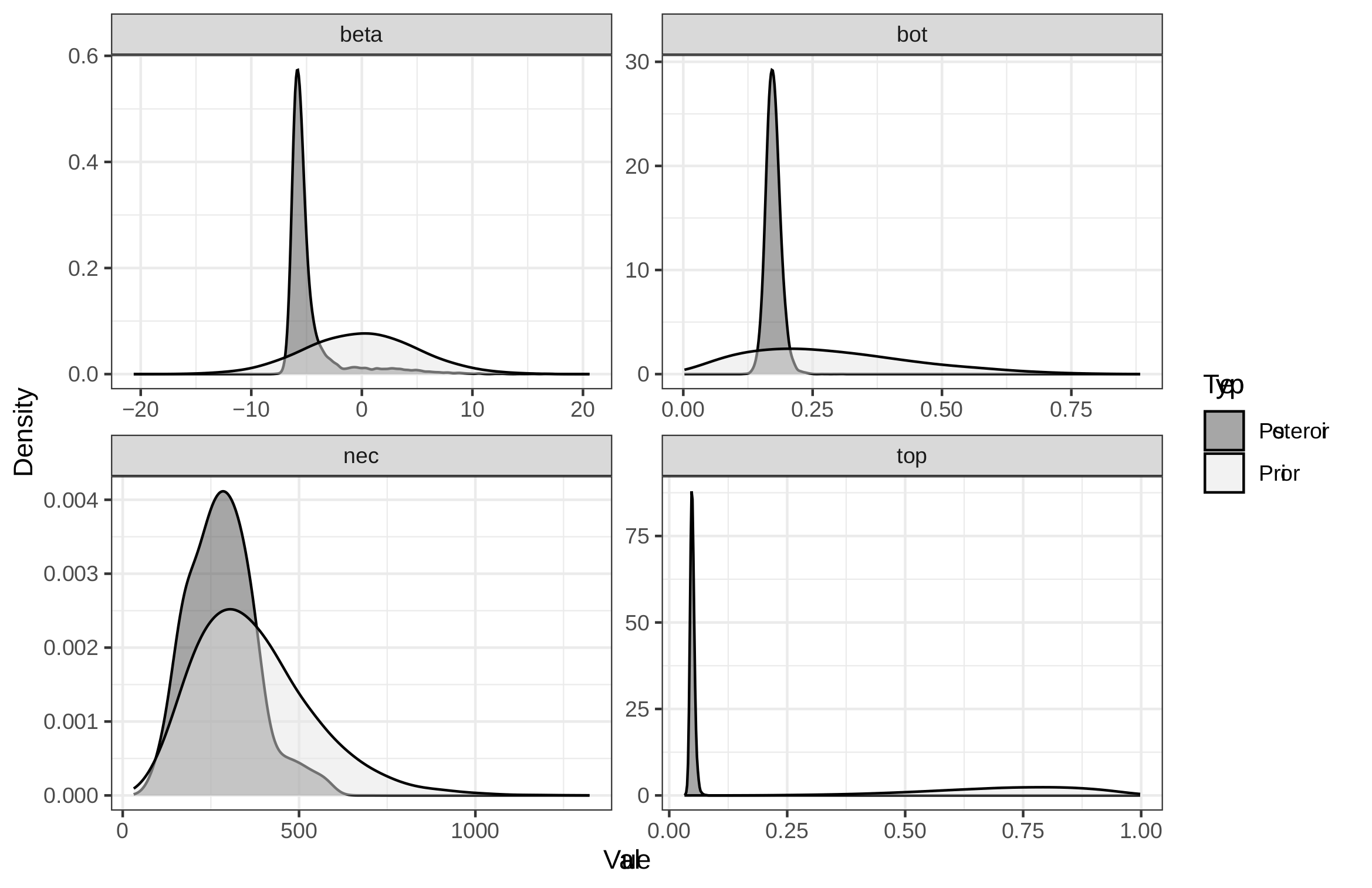


**Figure S4-1**. Prior and posterior distributions of the NEC model parameters (example: dataset 2023-09-26). The graphs compare the automatically assigned default priors used by the *bayesnec* package with the posterior parameter distributions obtained from the Markov chain Monte Carlo (MCMC) sampling. Shown are the parameters beta, bot, nec and top. Posterior distributions differ clearly from the priors, indicating that the data informed all parameters. Convergence diagnostics for this model run are shown in Figure S4-2.


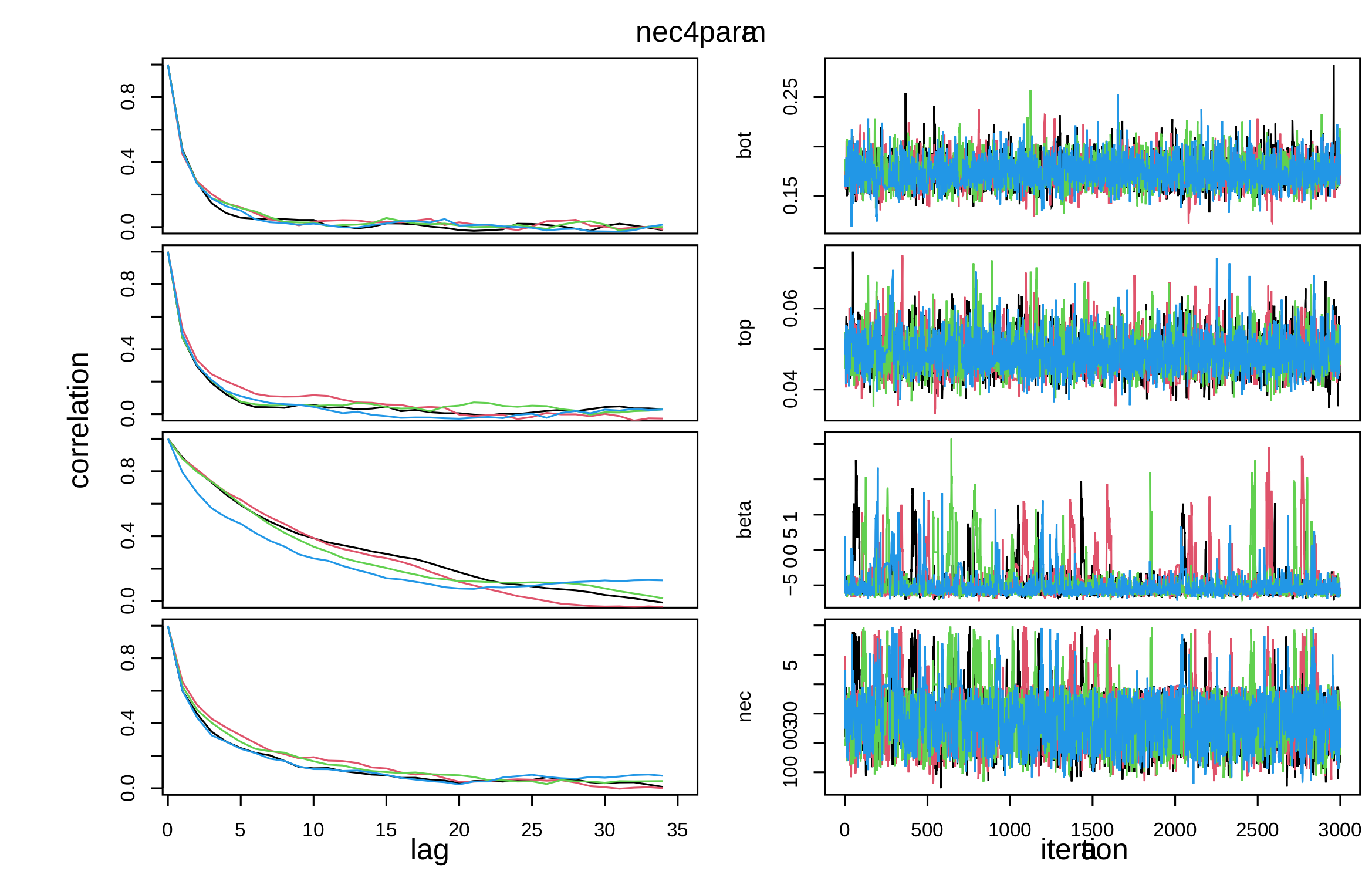


**Figure S4-2**. MCMC traceplots and autocorrelation functions for the nec4param model (example: dataset 2023-09-26). Four independent MCMC chains (15 000 iteration steps each) are shown for the parameters bot, top, beta and nec. The traceplots demonstrate good chain mixing without visible divergences, and the autocorrelation functions decay rapidly toward zero. Convergence for this model run was confirmed by *R̂* values < 1.01 and sufficiently large effective sample sizes.


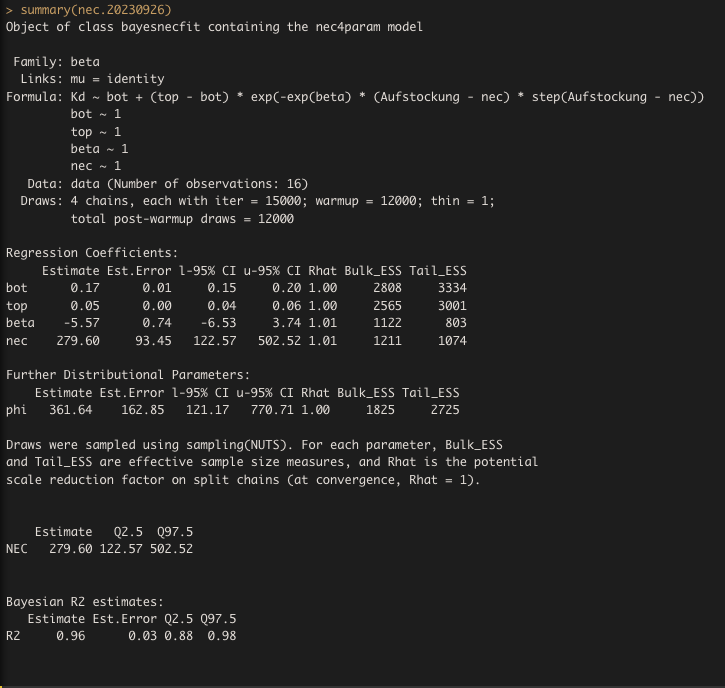


**Figure S4-3**. Output of the summary() function in bayesnec for one representative NEC model (sample from 2023-09-26). Shown are the regression coefficients with their 95% Bayesian credible intervals, distributional parameters, diagnostic statistics (effective sample sizes, Rhat), the NEC estimate with its 2.5-97.5% posterior quantiles, and the Bayesian R². This example illustrates how NEC values and their credible intervals used in the manuscript were obtained.

**S5. Lead speciation modelling**

**Table S5-1**. Input parameters used in the Visual MINTEQ (v4.0.1) speciation model. Dissolved Pb and EDTA originate from our measurements (median values for SP1 and SP2). All other parameters were derived from the monthly monitoring data (May 2023-June 2024) of the nearest NLWKN stations on the Innerste River (Heinde = SP1 analogue; Sarstedt = SP2 analogue).

| **Parameter** | **Unit** | **SP1 / Heinde value (median)** | **SP2 / Sarstedt value (median)** | **Source / Note** |
| --- | --- | --- | --- | --- |
| EDTA | µg L^‑1^ | 1.5 | 2.9 | This study |
| Pb (dissolved) | µg L^‑1^ | 0.70 | 0.69 | This study |
| Ca^2+^ | mg L^‑1^ | 94 | 100 | NLWKN |
| Mg^2+^ | mg L^‑1^ | 12 | 18 | NLWKN |
| Na^+^ | mg L^‑1^ | 43 | 60 | NLWKN |
| K^+^ | mg L^‑1^ | 3.4 | 6.4 | NLWKN |
| Cl^-^ | mg L^‑1^ | 71 | 99 | NLWKN |
| SO_4_^2-^ | mg L^‑1^ | 79 | 99 | NLWKN |
| Alkalinity  (acid capacity to pH 4.3) | mmol L^‑1^ | 3.6 | 3.9 | NLWKN |
| HCO_3_^-^  (MINTEQ input) | mg L^‑1^ | 220 | 240 | Derived from acid capacity |
| DOC | mg L^‑1^ | 1.7 | 2.1 | NLWKN |
| pH range | – | 4.0-10.0 | 4.0-10.0 | Modelling |
| pH increment | – | 0.25 | 0.25 | Modelling |
| EDTA scenario 1 | µg L^‑1^ | – | 20 | Moderate level |
| EDTA scenario 2 | µg L^‑1^ | – | 210 | NEC level |

**Table S5-2**. Modeled pH-dependent speciation of dissolved Pb (relative abundance) at SP2, calculated with Visual MINTEQ (v4.0.1).

| pH | Pb^+2^ | PbCl^+^ | PbOH^+^ | Pb(OH)_2_(aq) | Pb(OH)^3-^ | PbSO_4_(aq) | Pb(CO_3_)_2_^2-^ | PbCO_3_ (aq) | PbHCO_3_^+^ | PbEDTA^-2^ | PbHEDTA^-^ | PbH_2_EDTA(aq) | FA1-Pb(6)(aq) | FA2-Pb(6)(aq) |
| --- | --- | --- | --- | --- | --- | --- | --- | --- | --- | --- | --- | --- | --- | --- |
| 4,00 | 0,106 | 0,127 |  |  |  | 0,344 |  |  | 3,688 | 88,008 | 2,913 | 0,021 | 1,442 | 1,353 |
| 4,25 | 1,597 | 0,097 |  |  |  | 0,264 |  |  | 2,791 | 90,189 | 1,679 |  | 1,585 | 1,777 |
| 4,50 | 1,399 | 0,085 |  |  |  | 0,232 |  | 0,014 | 2,429 | 90,467 | 0,948 |  | 1,862 | 2,557 |
| 4,75 | 1,299 | 0,079 |  |  |  | 0,215 |  | 0,022 | 2,247 | 89,557 | 0,528 |  | 2,227 | 3,819 |
| 5,00 | 1,226 | 0,074 |  |  |  | 0,203 |  | 0,038 | 2,116 | 87,648 | 0,290 |  | 2,646 | 5,751 |
| 5,25 | 1,153 | 0,070 |  |  |  | 0,191 |  | 0,063 | 1,988 | 84,674 | 0,158 |  | 3,083 | 8,613 |
| 5,50 | 1,067 | 0,065 |  |  |  | 0,177 |  | 0,103 | 1,838 | 80,435 | 0,084 |  | 3,496 | 12,725 |
| 5,75 | 0,962 | 0,058 |  |  |  | 0,160 |  | 0,166 | 1,657 | 74,689 | 0,044 |  | 3,831 | 18,420 |
| 6,00 | 0,837 | 0,051 | 0,013 |  |  | 0,139 |  | 0,256 | 1,441 | 67,251 | 0,022 |  | 4,032 | 25,954 |
| 6,25 | 0,696 | 0,042 | 0,019 |  |  | 0,115 |  | 0,379 | 1,198 | 58,134 | 0,011 |  | 4,047 | 35,355 |
| 6,50 | 0,547 | 0,033 | 0,027 |  |  | 0,091 |  | 0,529 | 0,941 | 47,708 |  |  | 3,855 | 46,261 |
| 6,75 | 0,404 | 0,025 | 0,035 |  |  | 0,067 |  | 0,695 | 0,694 | 36,769 |  |  | 3,471 | 57,834 |
| 7,00 | 0,279 | 0,017 | 0,043 |  |  | 0,046 |  | 0,852 | 0,479 | 26,419 |  |  | 2,954 | 68,903 |
| 7,25 | 0,181 | 0,011 | 0,049 |  |  | 0,030 |  | 0,980 | 0,310 | 17,694 |  |  | 2,387 | 78,346 |
| 7,50 | 0,111 |  | 0,054 |  |  | 0,018 | 0,019 | 1,065 | 0,189 | 11,146 |  |  | 1,846 | 85,542 |
| 7,75 | 0,066 |  | 0,057 |  |  | 0,011 | 0,035 | 1,110 | 0,111 | 6,711 |  |  | 1,384 | 90,511 |
| 8,00 | 0,038 |  | 0,058 |  |  |  | 0,062 | 1,122 | 0,063 | 3,933 |  |  | 1,017 | 93,696 |
| 8,25 | 0,022 |  | 0,059 |  |  |  | 0,106 | 1,111 | 0,035 | 2,283 |  |  | 0,740 | 95,635 |
| 8,50 | 0,012 |  | 0,060 |  |  |  | 0,175 | 1,080 | 0,019 | 1,331 |  |  | 0,539 | 96,774 |
| 8,75 |  |  | 0,061 | 0,012 |  |  | 0,276 | 1,029 | 0,010 | 0,788 |  |  | 0,395 | 97,421 |
| 9,00 |  |  | 0,064 | 0,021 |  |  | 0,404 | 0,953 |  | 0,478 |  |  | 0,292 | 97,777 |
| 9,25 |  |  | 0,067 | 0,040 |  |  | 0,539 | 0,851 |  | 0,298 |  |  | 0,220 | 97,978 |
| 9,50 |  |  | 0,072 | 0,077 |  |  | 0,643 | 0,726 |  | 0,192 |  |  | 0,168 | 98,115 |
| 9,75 |  |  | 0,080 | 0,152 |  |  | 0,679 | 0,592 |  | 0,128 |  |  | 0,131 | 98,226 |
| 10,00 |  |  | 0,092 | 0,312 | 0,035 |  | 0,638 | 0,462 |  | 0,087 |  |  | 0,104 | 98,270 |
